# Rubella Virus Infections: A Bibliometric Analysis of the Scientific Literature from 2000 to 2021

**DOI:** 10.3390/healthcare10122562

**Published:** 2022-12-17

**Authors:** Hasan Ejaz, Hafiz Muhammad Zeeshan, Abid Iqbal, Shakil Ahmad, Fahad Ahmad, Abualgasim Elgaili Abdalla, Naeem Anwar, Kashaf Junaid, Sonia Younas, Ashina Sadiq, Muhammad Atif, Syed Nasir Abbas Bukhari

**Affiliations:** 1 Department of Clinical Laboratory Sciences, College of Applied Medical Sciences, Jouf University, Sakaka 72388, Saudi Arabia; 2 Department of Computer Sciences, National College of Business Administration and Economics, Lahore 54700, Pakistan; 3 Prince Sultan University, Riyadh 11586, Saudi Arabia; 4 Delta3T, Lahore 54700, Pakistan; 5 Allied Health Department, College of Health and Sport Sciences, University of Bahrain, Zallaq 32038, Bahrain; 6 School of Biological and Behavioural Sciences, Queen Mary University of London, London E1 4NS, UK; 7 HKU-Pasteur Research Pole, School of Public Health, LKS Faculty of Medicine, The University of Hong Kong, Hong Kong, China; 8 Department of Computer Science, Lahore Leads University, Lahore 54000, Pakistan; 9 Department of Pharmaceutical Chemistry, College of Pharmacy, Jouf University, Sakaka 72388, Saudi Arabia

**Keywords:** rubella infections, citation analysis, dimensions, R language, Biblioshiny, VOSviewer

## Abstract

Rubella virus (RuV) generally causes a mild infection, but it can sometimes lead to systemic abnormalities. This study aimed to conduct a bibliometric analysis of over two decades of RuV research. Medical studies published from 2000 to 2021 were analyzed to gain insights into and identify research trends and outputs in RuV. R and VOSviewer were used to conduct a bibliometric investigation to determine the globally indexed RuV research output. The Dimensions database was searched with RuV selected as the subject, and 2500 published documents from the preceding two decades were reviewed. The number of publications on RuV has increased since 2003, reaching its peak in 2020. There were 12,072 authors and 16,769 author appearances; 88 publications were single-authored and 11,984 were multi-authored. The United States was the most influential contributor to RuV research, in terms of publications and author numbers. The number of RuV-related articles has continued to increase over the past few years due to the significant rubella burden in low-income nations. This study will aid in formulating plans and policies to control and prevent RuV infections.

## 1. Introduction

Rubella virus (RuV) is an RNA virus and a member of the *Rubivirus* genus in the *Matonaviridae* family [1]. It has a positive-polarity single-stranded RNA genome roughly 10 kb in size, surrounded by capsid proteins arranged in a roughly spherical shape but not icosahedral, and an external bilayer lipid envelope. The RuV genome encodes viral nonstructural and structural proteins, including the capsid and envelope glycoproteins E1 and E2 [2,3]. Although RuV has only a single serotype and vaccination programs have significantly reduced RuV-infected cases, it continues to spread in countries with low vaccination coverage [4,5]. Approximately 30% of the global population remains unvaccinated against RuV and is at risk of infection [6]. RuV is a contagious airborne virus that causes asymptomatic, mild, or severe illness that manifests as fever, lymphadenopathies, and generalized skin rashes [7].

When infection occurs within the first eight weeks of pregnancy, RuV can have tragic and severe deleterious effects on the fetus, including malformations, premature delivery, and miscarriage [8]. Fetal malformation or congenital rubella syndrome (CRS) can affect multiple organs, resulting in permanent defects, including microcephaly, encephalopathy, heart defects, retinopathy, and deafness [9]. Approximately 100,000 cases of CRS occur worldwide each year [10,11]. However, the frequency of these cases may be higher than reported because vaccination coverage is only 70%. Therefore, establishing a rigorous and active survey program for rubella and its related congenital syndrome, as well as nationwide introduction of the rubella vaccine—particularly in developing countries—may be crucial strategies for decreasing the incidence of rubella infection and CRS [6]. 

Bibliometrics is a valuable tool for studying the scientific literature and output through the application of mathematical, statistical, computational, and other methodologies to help extract useful information [12]. Bibliometric analysis considerably increases the quality of literature reviews by offering a transparent, systematic, and repeatable review method. It can analyze the trend in a scientific topic, disclose gaps, and explore key research directions through in-depth investigations of databases, yielding fruitful results that policymakers and funding agencies can exploit to revise their strategies. Bibliometric analyses have been used to review the literature on COVID-19 [13], AIDS [14], hepatitis B virus [15], oncolytic virus [16], and zika virus [17]. Nevertheless, they have not yet been applied to the literature on RuV. This study aimed to use a bibliometric analysis to evaluate the current situation in RuV research in the literature, and to visualize the characteristics and trends in this research that will help to identify future prospects. Furthermore, we evaluated the year-by-year distribution of articles to identify the most productive institutes and countries, as well as the authors with the greatest influence.

## 2. Materials and Methods

### 2.1. Study Design

A bibliometric analysis was conducted in this study to considerably increase the quality of the literature review by offering a transparent, systematic, and repeatable review method. It allows for mapping study domains and influential work without subjectivity, which is essential for a holistic approach to the literature review process. The use of statistical and mathematical tools to analyze books and media communications is known as bibliometric analysis. Biblioshiny is a web-based application that provides a user interface for Bibliometrix for non-programmers. It facilitates researchers’ usage of Bibliometrix’s key features. To conduct a bibliometric analysis, various databases are available; each database has distinct characteristics and can offer various features. The most frequently used literature databases are PubMed and Dimensions, both of which are free. However, Web of Science and Scopus cover almost all disciplines, but are only available as subscription databases. Based on sources, authors, documents, and clustering by coupling, analytics and graphs are generated for four distinct levels of metrics. The three K-structures of knowledge can be analyzed using conceptual, intellectual, and social structures. R Studio was initially used to install and load the Bibliometrix R package. By typing Biblioshiny() into the R terminal, the Biblioshiny application was started. A number of tools made available by Bibliometrix enable investigators to conduct comprehensive bibliometric analyses [18,19,20,21]. It allows the display of multiple results in tables and graphs, which is a feature difficult to find in other software.

### 2.2. Data Sources

The bibliometric analysis was performed with RuV-centered research from 1 January 2000 to 31 December 2021. Articles from Dimensions were retrieved and evaluated using visualization tools Biblioshiny (K-Synth Srl, Naples, Italy) and VOSviewer (Leiden University, Leiden, The Netherlands).

### 2.3. Eligibility Criteria

**Figure 1:**
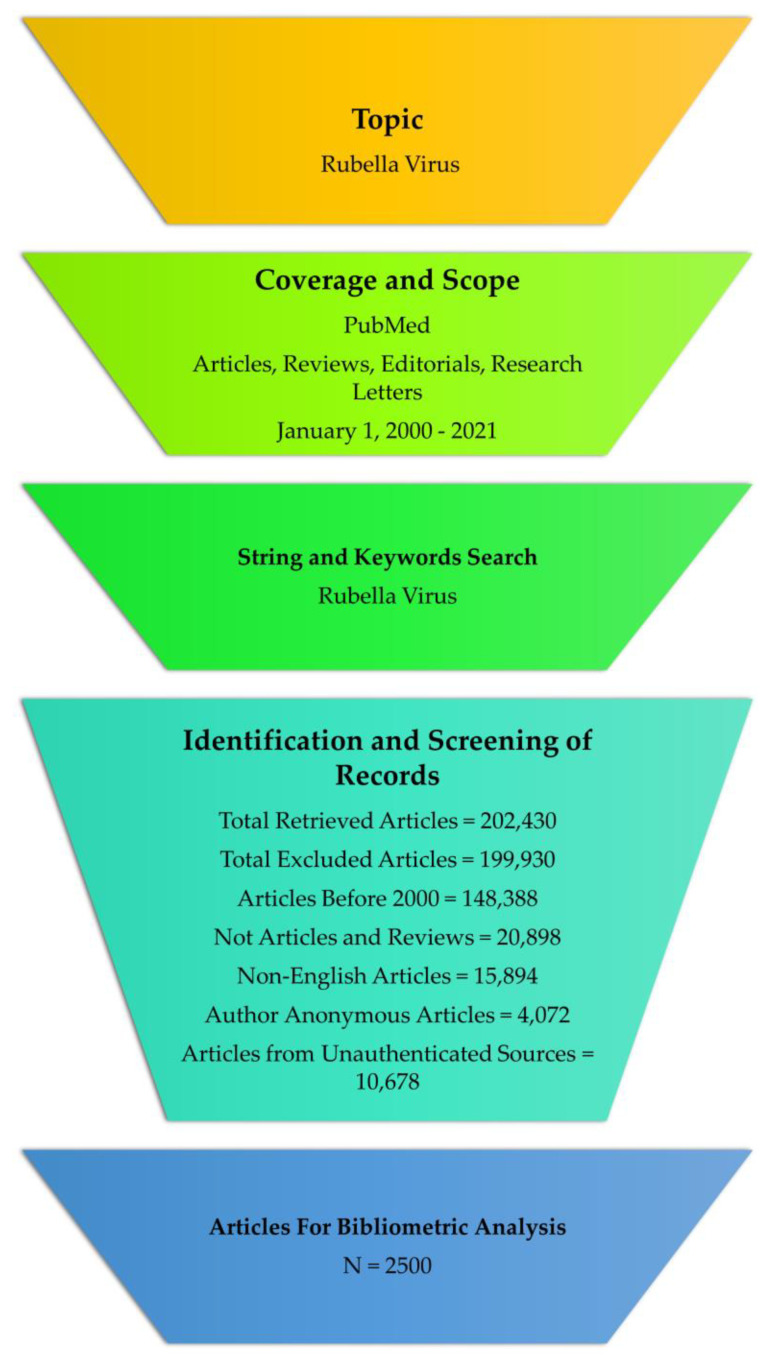
Procedure for selecting appropriate articles.

In this study, a standardized search approach was used for the bibliometric analysis based on the inclusion of the keyword “rubella virus” in the title, abstract, and keywords. The rubella virus data were collected from January 2000 to December 2021. The analysis included all related original articles, reviews, editorials, and research letters containing the keywords; however, abstracts, communications, and errata/corrections were excluded from the study. Figure 1 demonstrates the strategy for the published document selection.

### 2.4. Data Extraction

To avoid any bias in data collection, two independent investigators (Hafiz Muhammad Zeeshan and Fahad Ahmad) collected data on the same day as the search. After a conversation with a third investigator (Hasan Ejaz), the differences were discussed and resolved. The title of the article, authors’ names, times cited, citations per document, most-cited papers, year of publication, type of paper, countries/regions, institutions/organizations, and journal name were evaluated from the retrieved data.

### 2.5. Ethical Approval

As this study analyzed previously published research, no ethical approval was required. None of the authors of the studies included were contacted for more information about their respective publications.

### 2.6. Experimental Setup

The analysis was performed on a Lenovo Mobile Workstation (Levono, Morrisville, NC, USA) with an Intel Core i9 processor, NVIDIA RTX A5000 graphics card, 128 GB DDR4 memory, a 1 TB SSD hard drive, and Windows 11 Pro 64 as the operating system. We first installed the Bibliometrix package and loaded it into R Studio (Posit Corp, Boston, MA, USA). A .csv Excel file was uploaded to the Biblioshiny interface, as an Excel file is not the same as .csv Excel. The study also used Excel files (.csv) and portable network graphics files (.png) for the data analysis. The VOSviewer was used to present detailed information about RuV-based research, as well as for the extraction of other patterns.

### 2.7. Data Analysis

For the most productive and influential authors, various factors such as authorship pattern, degree of collaboration, and year-by-year groupings of articles were used in the analysis of their papers in PubMed. According to the analysis, between 2000 and 2021, 2500 documents that matched the study criteria were published in Dimensions (Figure 1).

## 3. Results

### 3.1. Characteristics of the Studies on Rubella Virus 

The primary information about studies on RuV on Dimensions from 2000 to 2021 is shown in Table 1. A total of 2500 publications and 31,897 citations were collected from Dimensions. The average number of citations per publication based on the established criteria for the 749 most significant sources (articles, reviews, editorials, and research letters) was 12.76. According to the results, there were 12,072 authors and 16,769 author appearances, of which 88 were single-authored documents and 11,984 were multi-authored publications.

### 3.2. Distribution of Publication Year of Rubella Virus-Based Studies

**Figure 2:**
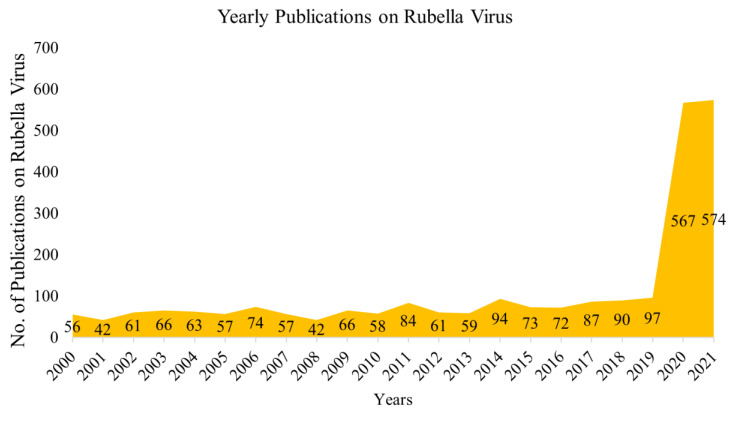
The trend in annual scientific publications on rubella virus.

Figure 2 indicates that 2020 was the year in which the most articles were published, with a total of 566, and 2001 was the least prolific, with only 24 articles published in research journals.

### 3.3. Distribution of Types of Documents on Rubella Virus-Based Studies 

**Figure 3:**
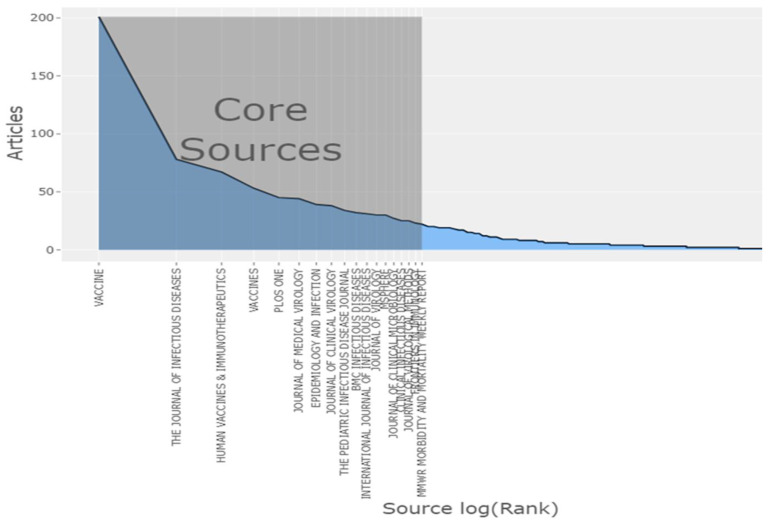
Source distribution by Bradford’s law.

The number of articles by Bradford’s law zones in core sources was found to be 844 with 15,341 citations, and the number of core + zone 2 sources was 1675 with 29,473 citations, as shown in Figure 3.

### 3.4. Characteristics of the Most Cited Studies on Rubella Virus 

**Figure 4:**
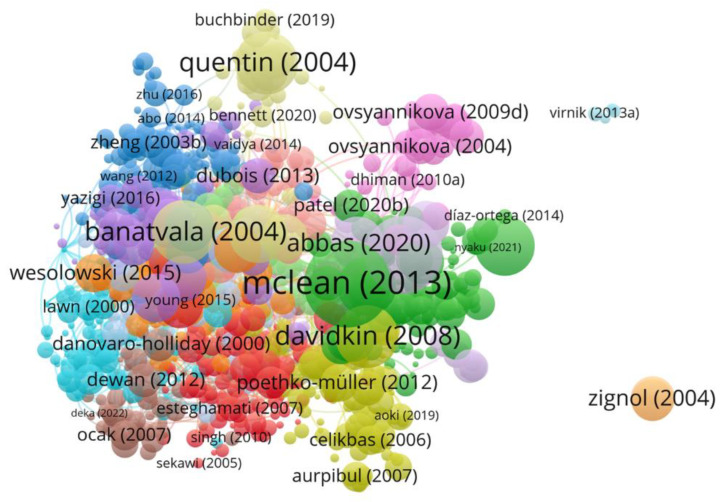
Most cited studies on rubella virus.

The articles were arranged in descending order by frequency of citation. The top three most cited papers were further analyzed for their citations. The top three locally cited documents were Banatvala, J. E., & Brown, D. W. (2004). Rubella. The Lancet, 363(9415), 1127–1137; Lambert, N., Strebel, P., Orenstein, W., Icenogle, J., & Poland, G. A. (2015). Rubella. The Lancet, 385(9984), 2297–2307; and Davidkin, I., Jokinen, S., Broman, M., Leinikki, P., & Peltola, H. (2008). Persistence of measles, mumps, and rubella antibodies in an MMR-vaccinated cohort: a 20-year follow-up. The Journal of infectious diseases, 197(7), 950–956, with 96, 92, and 91 local citations and 255, 214, and 247 global citations, respectively (Figure 4).

The top three globally cited documents were Amanna, I. J., Carlson, N. E., & Slifka, M. K. (2007). Duration of humoral immunity to common viral and vaccine antigens. New England Journal of Medicine, 357(19), 1903–1915: Desailloud, R., & Hober, D. (2009). Viruses and thyroiditis: an update. Virology journal, 6(1), 1–14; Pollard, A. J., & Bijker, E. M. (2021). A guide to vaccinology: from basic principles to new developments. Nature Reviews Immunology, 21(2), 83–100, with 874, 313, and 260 total citations and 54.62, 22.35, and 86.66 total citations per year.

The top three locally cited references were Frey, T. K. (1994). Molecular biology of rubella virus. Advances in virus research, 44, 69–160; McLean, H. Q., Fiebelkorn, A. P., Temte, J. L., & Wallace, G. S. (2013). Prevention of measles, rubella, congenital rubella syndrome, and mumps, 2013: summary recommendations of the Advisory Committee on Immunization Practices (ACIP). Morbidity and Mortality Weekly Report: Recommendations and Reports, 62(4), 1–34; and Miller, E., Cradock-Watson, J., & Pollock, T. (1982). Consequences of confirmed maternal rubella at successive stages of pregnancy. The Lancet, 320(8302), 781–784, with 119, 103, and 101 citations, respectively.

### 3.5. Distribution of Authors of Rubella Virus-Based Publications

The top three authors who published the highest number of studies were Gregory A. Poland, Joseph Icenogle, and Inna G. Ovsyannikova, with 53, 44, and 42 articles and 11.06, 9.40, and 7.39 article fractions, respectively. The top three authors with the highest number of local citations were Inna G. Ovsyannikova, Stanley A. Plotkin, and Elizabeth Miller with 313, 270, and 234 citations, respectively. With respect to production over time, Gregory A. Poland, Joseph Icenogle, and Inna G. Ovsyannikova were the most prolific in 2000–2021.

**Figure 5:**
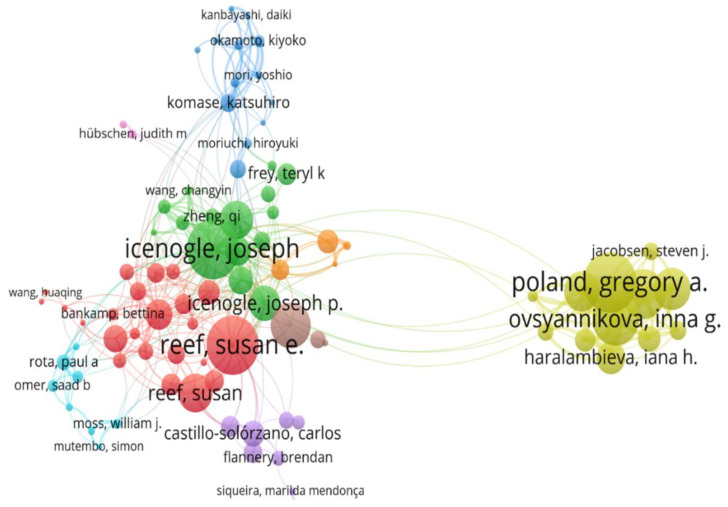
Outstanding authors of rubella virus research publications.

The three authors with the highest impact were Gregory A. Poland, Joseph Icenogle, and Inna G. Ovsyannikova, with an h-index of 26, 21, and 23; a g-index of 39, 35, and 34; m-index of 1.18, 0.95, and 1.211; total number of citations of 1655, 1280, and 1226; and publication year starting from 2001, 2001, and 2004, respectively. The above findings are shown in Figure 5.

### 3.6. Source Impact

**Figure 6:**
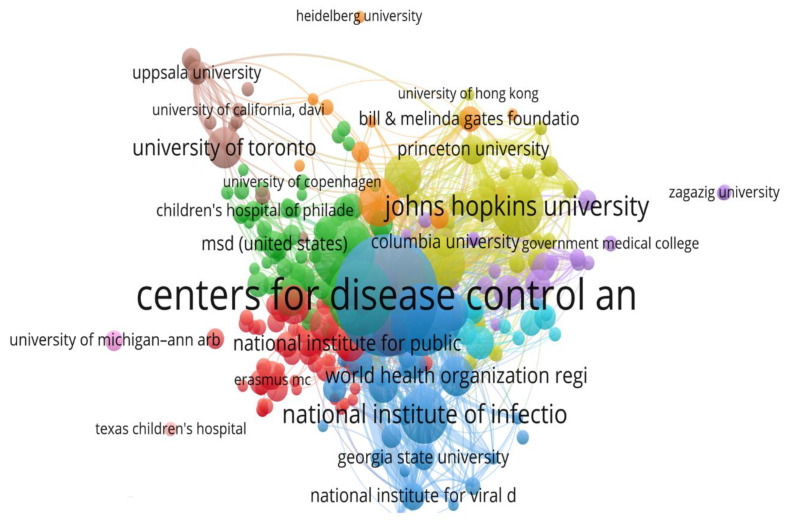
Outstanding institutions involved in rubella virus research and their accomplishments.

The majority of the studies included in the analysis were authored by researchers from the following three institutions (Figure 6): the Centers For Disease Control And Prevention (105 articles)—the national public health agency of the United States; the Mayo Clinic (63 articles)—a nonprofit academic medical center from the United States, with a focus on integrated healthcare, education, and research; and Public Health England (59 articles) to safeguard and enhance health and lessen health disparities—an executive agency of the Department of Health and Social Care in England, established in 2013.

### 3.7. Distribution of Countries Involved in Rubella Virus-Based Studies

Most of the studies were published by three countries: Japan (n = 303), Germany (n = 271), and the United Kingdom (n = 262). Despite the reality that East and South Asia and Africa bear a disproportionate share of the worldwide burden of rubella, their research output was limited. This should prompt health organizations to reconsider their support for and funding of RuV research in disease-endemic nations in order to create effective control and prevention strategies.

**Figure 7:**
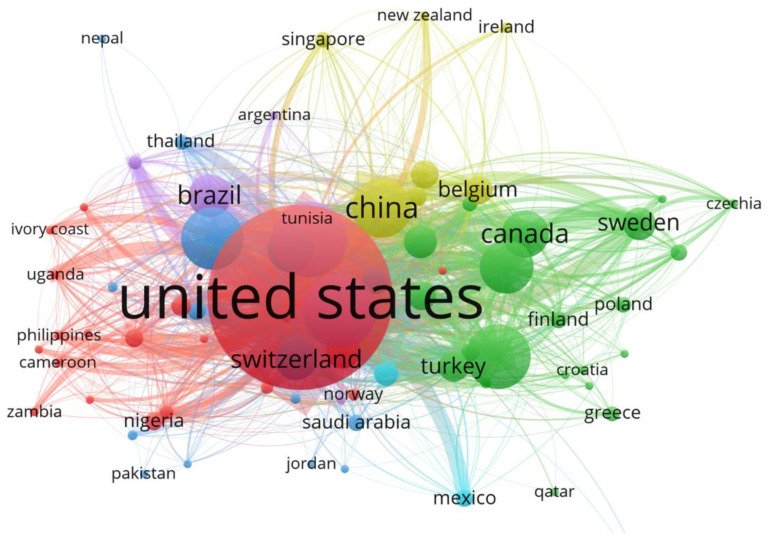
Outstanding countries contributing to rubella virus research.

The three outstanding countries whose publications on RuV received the highest number of citations were the United States (n = 13,077), the United Kingdom (n = 3001), and Germany (n = 2332). The top three countries of corresponding authors of publications were the United States, Japan, and India, with 561, 149, and 123 articles; a frequency of 0.22, 0.06, and 0.06; 267, 75, and 68 inter-country publications; and 294, 74, and 55 intra-country publications, respectively (Figure 7).

### 3.8. Distribution of Sources of Rubella Virus-Based Studies

The top three relevant sources were the journals *Vaccine* from Elsevier, *The Journal of Infectious Diseases* (*J. Infect. Dis.*) from Oxford University Press, and *Human Vaccines & Immunotherapeutics* from Taylor & Francis, with 201, 78, and 67 articles, respectively.

The sources with the highest local citations (from reference lists) were *Vaccine* from Elsevier, *J. Infect. Dis.*, and *The Lancet* from Elsevier with 4841, 3131, and 2303 articles, respectively.

The top three sources with respect to impact were *Vaccine* from Elsevier, *J. Infect. Dis.*, and *Human Vaccines & Immunotherapeutics* from Taylor & Francis, with an h-index of 35, 30, and 11; a g-index of 51, 50, and 18; an m-index of 1.52, 1.50, and 0.91; total number of citations of 3977, 2746, and 467; and publication year starting from 2000, 2003, and 2011, respectively.

**Figure 8:**
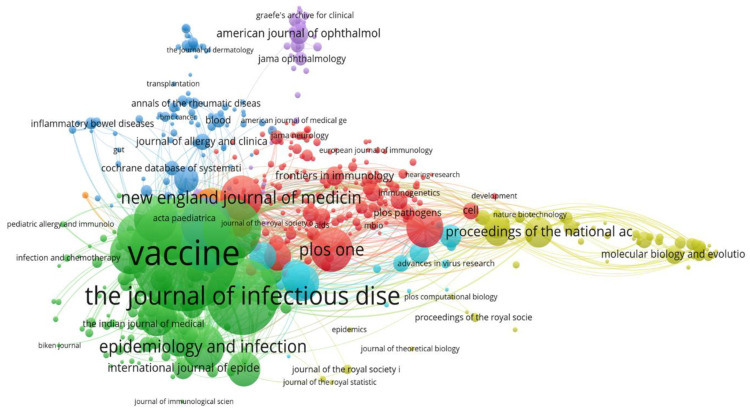
Outstanding sources contributing to rubella virus research.

The dynamics of the top three journals with respect to occurrences per year were: *J. Infect. Dis.*, *BMC Infectious Diseases* from Springer, and *Vaccines* from MDPI, with 76, 33, and 60 occurrences to date in comparison to the previous year’s 76, 28, and 56 occurrences, respectively (Figure 8).

### 3.9. Distribution of Keywords in Rubella Virus-Based Studies

**Figure 9:**
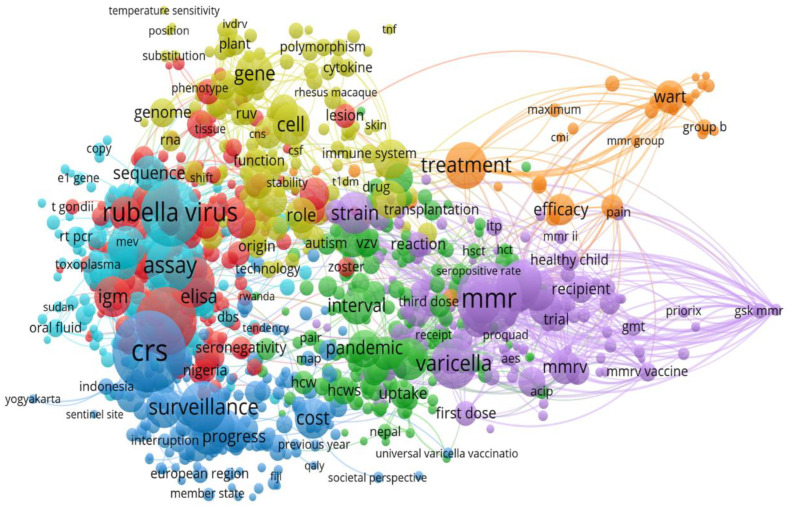
Dominant keywords in publications on rubella virus research.

Overall, rubella, measles, and vaccination were the top three keywords used in the studies with 1417, 590, and 395 occurrences, respectively (Figure 9).

## 4. Discussion

This bibliometric analysis comprehensively described the research on rubella, a widespread disease in both industrialized and developing nations. Researchers worldwide have studied the detection, control, treatment, and avoidance of RuV. To our knowledge, no bibliometric analysis has been undertaken to date on the 2500 papers based on RuV research, despite the relevance of bibliometrics as a means of examining research volume, orientations, and collaborations between academics and medical practitioners. In recent decades, an increasing number of studies on the prevalence of RuV antibodies in various human groups have been published. In some 1.3 industrialized nations, RuV cases have surged by as much as 79% in recent years [22,23]. Different factors, including assays that detect anti-RuV IgG antibodies and geographical location, may contribute to the variation in the occurrence of RuV [24,25].

This study was undertaken for a citation analysis and its primary characteristics. In recent years, there has been a continuous increase in the number of RuV-related papers due to the global burden of this disease. Understanding the characteristics of published RuV research may be advantageous for multiple reasons. Our research revealed that the 2500 studies in Dimensions were referenced 31,897 times. The average number of citations per publication was 12.76, showing their significance and impact. Three studies were mentioned more than 1100 times in the scholarly literature. Previous research analyzing the RuV literature indicated a consistent growth in RuV-related publications over the past few decades. Using Web of Science, Deqiao Tian undertook a comprehensive literature review of pathogens, including health-threatening pathogens and biodefense-associated pathogens, and provided recommendations for future research on RuV [26]. Japan, the United Kingdom, and China were the most prolific publishers of studies on RuV. The United States, the United Kingdom, and Germany were the top three countries publishing on RuV in general. Though Tian’s study indicated a plateau research trend for RuV, our results revealed a high trend of publications (Figure 2). The difference may be due to the different databases or the keywords used for searching the literature.

Our study revealed that the highest number of corresponding authors of articles on RuV were from the United States, Japan, and India. This trajectory in publishing is inconsistent with the worldwide global distribution of the disease, as it is more prevalent in low- and middle-income nations, where it appears as both epidemics and sporadic infections. This study emphasized the need to establish research connections between experts from rich and resource-limited nations [27]. To prevent and control disease outbreaks, it is necessary to encourage researchers, particularly those from countries with low resources, to devote more time to RuV research by providing them with technical and financial support. According to the Bradford analysis, there are 17 journals in the core zone. This indicates that only 17 journals contributed 50.38% of the published material, representing a significant concentration of publications by a limited number of journals. The importance of journal quality and scope in the communication of research to all constituencies cannot be overstated. The journals most prolific in publishing articles on RuV are *Vaccine*; *Human Vaccines & Immunotherapeutics*; and *The Journal of Infectious Diseases*. *Vaccine* and *Human Vaccines & Immunotherapeutics* have published the highest number of articles on RuV. The *Vaccine* journal features a hybrid publication mode, meaning writers have the option of publication with subscription or open access, while *Human Vaccines & Immunotherapeutics* and *The Journal of Infectious Diseases* are open access with different waive options for authors from low-income countries, which greatly aids academics from developing nations to disseminate local material to a worldwide readership [27].

This emphasizes the necessity of other journals adopting subscription or fee-waiver policies in order to attract high-quality publications from poorer nations [28]. Local research of high quality may be conducted in low- and middle-income nations, but it is never published in international journals and is not distributed to the public for lack of funding. This research serves as a valuable resource for medical virologists, epidemiologists, policymakers, academicians, and researchers. The report provides an overview of the evolution of the rubella virus and the direction of research into its eradication.

The data reported in this paper may be valuable for researchers and scientists in focusing their study aims on more relevant domains within the field of RuV research. In addition, it can assist academics and teachers by providing high-quality bibliographic references for educational purposes. Healthcare providers and parents need to be targeted with adequate information on RuV. Research on RuV should be encouraged, and health practitioners should be involved in collaborative research for public awareness. This study has several limitations, such as the fact that we only retrieved data from papers indexed in Dimensions; articles from other databases were not analyzed, which may have affected the conclusion. In addition, the quality of the most cited publications was not evaluated, which may have affected the interpretation of the results.

## 5. Conclusions

This study discussed the most cited articles on RuV and their authors, institutions, and countries in the context of the RuV research output over the past two decades. In both developing and developed nations, RuV is regarded as the cause of one of the most contagious person-to-person airborne viral infections. Only two vaccines against RuV are available, MMR and MMRV, the latter containing weakened measles, mumps, rubella, and varicella viruses. The majority of studies on RuV are published by researchers from wealthy nations, and the United States was the most influential contributor. This study highlights the need to develop research partnerships between scholars from industrialized and low-income nations. To forestall and manage the spread of rubella, it is vital to motivate researchers, especially those from countries with few resources, to concentrate more on RuV research by providing them with technical and financial support. This study will also aid in formulating plans and policies for the prevention and control of RuV. The most dominant term used in these studies was vaccination against rubella virus, which highlights the significance of immunization, especially in infants. More importantly, it can guide decision-making strategies in medical services from a public health perspective.

## Figures and Tables

**Table 1:** Information about studies on RuV on Dimensions from 2000 to 2021.

Description	Results
Time span	2000–2021
Sources (journals, books, etc.)	773
Documents (articles, reviews, editorials, and research letters)	2500
Keywords plus (ID)	1
Authors’ keywords (DE)	1
Average citations per document	16.63
Authors	11,782
Author appearances	16,308
Authors of single-authored documents	125
Authors of multi-authored documents	11,669
Single-authored documents	125
Documents per author	0.212
Authors per document	4.71
Co-authors per document	6.52
Collaboration index	4.93

## Data Availability

All data generated during the study are presented in this paper.
